# A Double-Hybridization Approach for the Transcription- and Amplification-Free Detection of Specific mRNA on a Microarray

**DOI:** 10.3390/microarrays5010005

**Published:** 2016-02-23

**Authors:** Michaela Haider, Thomas Haselgrübler, Alois Sonnleitner, Fritz Aberger, Jan Hesse

**Affiliations:** 1 Center for Advanced Bioanalysis GmbH, Gruberstrasse 40-42, 4020 Linz, Austria; michaela.haider@cbl.at (M.H.); thomas.haselgruebler@cbl.at (T.H.); alois.sonnleitner@cbl.at (A.S.); 2 Department of Molecular Biology, University of Salzburg, 5020 Salzburg, Austria; fritz.aberger@sbg.ac.at

**Keywords:** microarray, mRNA detection, enzyme-free, gene expression, fluorescence microscopy

## Abstract

A double-hybridization approach was developed for the enzyme-free detection of specific mRNA of a housekeeping gene. Targeted mRNA was immobilized by hybridization to complementary DNA capture probes spotted onto a microarray. A second hybridization step of Cy5-conjugated label DNA to another section of the mRNA enabled specific labeling of the target. Thus, enzymatic artifacts could be avoided by omitting transcription and amplification steps. This manuscript describes the development of capture probe molecules used in the transcription- and amplification-free analysis of *RPLP0* mRNA in isolated total RNA. An increase in specific signal was found with increasing length of the target-specific section of capture probes. Unspecific signal comprising spot autofluorescence and unspecific label binding did not correlate with the capture length. An additional spacer between the specific part of the capture probe and the substrate attachment site increased the signal significantly only on a short capture probe of approximately 30 nt length.

## 1. Introduction

The majority of methods for mRNA expression analysis, such as quantitative polymerase chain reaction (qPCR) [1], RNA-sequencing [2,3] and microarray analysis [3], start with the reverse transcription (RT) of mRNA into complementary DNA (cDNA). The RT step, however, can introduce quantification bias due to variable efficacy and fidelity of the RT enzyme. For instance, Ståhlberg *et al.* [4] found RT yields varying up to 100-fold dependent on the RT enzyme and the target gene. Moreover, with oligo(dT) primers, which are often used for priming the RT reaction, only polyadenylated RNA molecules are reverse-transcribed, while RNA species without poly(A) tail [5] are not available for analysis [6]. Furthermore, cDNAs often do not represent full-length mRNA molecules, which can limit the subsequent analysis to certain mRNA sections [6]. Reverse transcription can also introduce artifacts due to template switching [7,8], primer-independent cDNA synthesis [9] and DNA-dependent DNA polymerase activity [10]. Being the starting point for exponential quantification, the RT step is therefore one of the main contributors to technical variation in RT-qPCR analysis [11,12].

Exponential amplification of nucleic acids with PCR is moreover inherently error-prone, for instance, by introduction of Taq DNA polymerase errors and the formation of chimeric and heteroduplex molecules [13]. Both PCR and linear amplification based on *in vitro* transcription with T7 RNA polymerase can moreover introduce amplification bias [14,15]. Single-molecule fluorescence *in situ* hybridization (smFISH) is a widely used technology for direct detection of mRNA without prior reverse transcription or target amplification [16]. While being a highly specific and sensitive method [16], smFISH assays are generally limited to multiplexing up to a dozen transcripts by virtual color barcoding [17,18]. In contrast, microarray technology allows for the parallel analysis of tens of thousands of genes due to spatial separation of individual target genes.

The NanoString nCounter gene expression assay is another hybridization-based system which enables the detection of target mRNA without prior reverse transcription. The protocol has a multiplexing capability of several hundred target genes per sample [19]. Analysis of single cells, however, still requires reverse transcription and linear PCR amplification prior to hybridization [20,21].

Our group has recently shown an enzyme-free double-hybridization assay for the specific detection of short DNA and RNA oligonucleotides on a microarray with single-molecule sensitivity [22]. In the present report, we optimized this approach to enable the analysis of endogenous mRNA of a housekeeping gene contained in isolated total RNA. This report presents design improvements for capture probes used in the transcription- and amplification-free analysis of *RPLP0* mRNA on a microarray, which may be also applied for the development of probes for other genes.

## 2. Materials and Methods

If not otherwise stated, chemical reagents were purchased from Sigma-Aldrich (Vienna, Austria). Denatured ethanol (EtOH) was purchased from Carl Roth (Vienna, Austria), deionized formamide was purchased from PanReac AppliChem (Darmstadt, Germany), EtOH absolute was purchased from AustrAlco (Spillern, Austria), and UltraPure salmon sperm DNA solution was purchased from Thermo Fisher Scientific (Vienna, Austria). Cell culture media, media supplements and antibiotics were purchased from Thermo Fisher Scientific. All buffers were prepared using ultrapure water (Milli-Q, 18.2 MΩ·cm at 25 °C, Merck Millipore, Vienna, Austria) and filtered through sterile 0.22 µm polyvinylidene fluoride (PVDF) syringe filters (Carl Roth, Austria). Surfaces and instruments were wiped with the surface decontaminant RNase Away (Carl Roth, Austria) prior to work.

### 2.1. Panc-1 Cell Culture

As described elsewhere [23], the human pancreas epithelioid carcinoma cell line Panc-1 (ATCC CRL-1469) was maintained in Dulbecco’s Modified Eagle’s Medium (DMEM) supplemented with 10% fetal bovine serum (FBS), 100 units/mL penicillin and 100 μg/mL streptomycin (PenStrep) in a humidified atmosphere at 37 °C and 5% CO_2_. The cells were routinely passaged at 80%–90% confluence twice a week.

### 2.2. Double-Hybridization Principle

**Figure 1:**
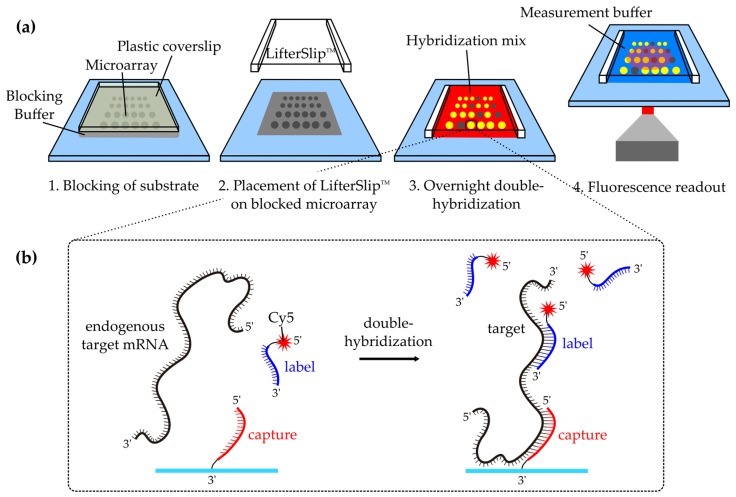
Assay principle: (**a**) Amino-modified microarray capture probes were spotted on epoxy-coated glass substrates. After washing away excess capture probes, the reactive surface of the substrates was subsequently blocked with 1% (*w/v*) sodium dodecyl sulfate and blocking buffer (grey). An 18 × 18 mm^2^ glass coverslip with raised edges (LifterSlip™) was placed atop the microarray. Then hybridization mix (red) was injected, mRNA secondary structures were denatured and overnight double-hybridization was performed. The next day, unbound compounds were washed away and the microarray was imaged using a fluorescence microscope; (**b**) Double-hybridization was based on immobilization of target mRNA on a spotted complementary DNA probe (capture). The mRNA was labeled by hybridizing a complementary Cy5-tagged DNA probe (label) to another section of the target mRNA.

As illustrated in Figure 1b, the double-hybridization approach was based on specific immobilization of target mRNA molecules by hybridization to spotted complementary DNA capture probes on a microarray. Fluorescent labeling of target mRNA was achieved by hybridizing 5′‑Cyanine 5 (Cy5)‑labeled complementary DNA probes (subsequently denoted as labels) to a different section of the mRNA. This approach has been shown before for the ultra-sensitive detection of *in vitro* synthesized short DNA and RNA molecules in a dynamic microfluidic chip [22].

### 2.3. Probe Design

All capture and label probes were designed *in silico* based on GenBank reference sequences [24]. Probe sequences were chosen such that cross-hybridization events and secondary structures were minimized. Moreover, a GC content between 40% and 60% should be obtained. Lyophilized probes were purchased from Microsynth (Balgach, Switzerland). Label probes were dissolved in Tris-EDTA (TE) buffer (1 mM ethylenediaminetetraacetic acid (EDTA), 10 mM Tris; pH 7.0) and kept in the dark. 3′‑Amino‑modified capture probes were dissolved in TE buffer (pH 8.0) according to the manufacturer’s instructions. All oligonucleotides were stored as single-use aliquots at −80 °C. The sequences of all probes are stated in Table S1.

### 2.4. Isolation of Total RNA for Microarray Analysis

Total RNA for microarray analysis was isolated from Panc-1 cells with an RNeasy Mini Kit (Qiagen, Vienna, Austria) according to the manufacturer’s instructions. In brief, 3 × 10^6^ Panc-1 cells were lysed by adding 350 µL lysis buffer (RLT buffer) and thoroughly resuspending. After mixing the homogenized lysate with 350 µL of 70% EtOH absolute, the solution was transferred to an RNeasy spin column placed in a collection tube. Following centrifugation (15 s, 10,000× *g*), the flow-through was discarded and the column was washed sequentially with 700 µL RW1 buffer and 2 × 500 µL RPE buffer. Purified total RNA was subsequently eluted in 30 µL nuclease-free water.

### 2.5. DNA Microarray Fabrication

A Microgrid II contact-printer and SMP2 stealth pins (for a spot diameter of approximately 62.5 µm) (ArrayIt, Sunnyvale, CA, USA) were used to print 3′-amino-(CH_2_)_7_-modified probes (subsequently denoted as captures) on epoxy-functionalized substrates (NEXTERION Slide E, 50 × 24 × 0.175 mm^3^, Schott Technical Glass Solution GmbH, Jena, Germany). Therefore, the capture probes were diluted in spotting buffer (1.5 M betaine monohydrate, 0.001% (*w/v*) CHAPS, 0.005% (*w/v*) sodium dodecyl sulfate (SDS), 4× saline sodium citrate buffer (SSC)) to a working concentration of 15 µM. All substrates were washed with denatured EtOH and nuclease-free water prior to spotting. Following spotting, the substrates were allowed to rest at room temperature overnight for covalent attachment of the amine-modified capture probes to the epoxy-functionalized substrate. Excess capture probes were washed away by dipping the substrate into three beakers of washing buffer (0.1% (*w/v*) SDS, 1× SSC) and three beakers of nuclease-free water.

### 2.6. Double-Hybridization of RPLP0 mRNA

As depicted in Figure 1, spotted substrates were incubated with 50 µL 1% (*w/v*) SDS, 50 µL blocking buffer (100 mM ethanolamine, 0.1% (*w/v*) SDS, 100 mM Tris; adjusted to pH 9.0), and 50 µL washing buffer at 40 °C for 10 min each prior to hybridization. During incubation, all solutions were covered with plastic coverslips (22 × 22 mm^2^, Cole-Parmer). All incubation steps were performed in a hybridization cassette (ArrayIt, Sunnyvale, CA, USA) that contained water-filled reservoirs to prevent evaporation of buffers. Afterwards, the substrates were dipped into a beaker of nuclease-free water to get a clean and dry surface. A LifterSlip™ (Science Services, Munich, Germany) with a volume of 7.6 µL (18 × 18 mm^2^) was placed atop of the microarray. After filling the LifterSlip™ with hybridization mix (HM), the hybridization cassette was closed and heated to 70 °C for 5 min to denature mRNA secondary structures. The HM comprised either 100 ng/μL isolated total Panc-1 RNA or the equal volume of nuclease-free water for mock hybridization experiments, as well as 1% (*w/v*) SDS, 3.5 × SSC, 1 mM EDTA, 50% (*v/v*) deionized formamide, 100 μg/mL UltraPure salmon sperm DNA solution, 100 pM Control(+) label and 100 pM Control(+) RNA. The *RPLP0*-specific labels that were used in the respective experiments are listed in Table 1. Overnight incubation at 40 °C in the dark allowed for double-hybridization of *RPLP0* mRNA to complementary capture and label probes.

### 2.7. Fluorescence Microscopy

Imaging was performed on an Axiovert 200 inverted microscope (Zeiss, Vienna, Austria) equipped with a motorized scanning stage (Scan IM 120 × 100, Märzhäuser, Wetzlar, Germany) and a CCD camera (CoolSnap HQ, Photometrics, Tucson, AZ, USA). Fluorescence imaging was performed in time delay and integration (TDI)‑mode [25] using a 642 nm diode laser (iBeam, Toptica Photonics AG, Gräfelfing, Germany) and 111 ms effective illumination time. Samples were illuminated through a long-pass filter (OG-550, Schott, Mainz, Germany) and a high-NA 100 × objective (α-Plan FLUAR 100 × 1.45 oil, Zeiss, Vienna, Austria). The obtained fluorescence signals were separated from excitation light by a dichroic beamsplitter (Q660LP, Chroma, Bellows Falls, VT, USA) and an emission filter (HQ700/75M, Chroma) before being imaged on the CCD camera. The microscope setup was further equipped with a focus hold system, which kept the distance from objective to sample constant [26,27].

Prior to imaging, the LifterSlip™ was removed from the microarray and unbound components were washed away by shaking the hybridized coverslip in 30 mL washing buffer (preheated to 37 °C) for 5 min. To prevent drying of the microarray surface during fluorescence measurement, the substrates were covered with measurement buffer (1% (*w/v*) SDS, 3.5 × SSC, 1 mM EDTA) filled into LifterSlip™ coverslips. All microarrays were heated to 42 °C using an objective heater (TempControl 37-2 digital, PeCon, Erbach, Germany) during measurement to avoid precipitation of the measurement buffer.

### 2.8. Data Analysis

Analysis of the average spot brightness was performed with MATLAB (MathWorks^TM^ Inc., Natick, MA, USA) as previously described [22]. In brief, for each spot, a sub-image was extracted from the raw data for further processing. To determine the average net intensity of the microarray spots, the local background adjacent to the respective spot was subtracted from the mean fluorescence intensity of the whole spot sub-image. Statistical analysis was conducted in SigmaPlot 12.0 (Systat Software, Inc., Washington, WA, USA). A one-way ANOVA was applied to test for statistically significant differences of net spot intensities. A *p* value of < 0.05 was considered to be statistically significant.

## 3. Results

### 3.1. Label Probe Optimization

The assay optimization was based on detection of the large ribosomal protein P0 (*RPLP0*). The lengths of the initial label and capture probes were based on a recent publication of our group dealing with double-hybridization of nucleic acids in a microfluidic chip [22].

First, the label probe concentration for double-hybridization analysis of *RPLP0* mRNA in 100 ng/µL isolated total Panc-1 RNA was optimized. Therefore, a *RPLP0* label dilution series was conducted with 100 pM–100 nM Label_33nt probe concentration while keeping the RNA concentration constant. As shown in Figure S1, the maximum specific *RPLP0* signal was obtained with a label concentration of 10 nM, wherefore this label concentration was used in all subsequent experiments. Then the effect of label probe elongation was tested. Signal intensities obtained using a 29 nt long *RPLP0*-specific capture probe as well as three label probes with different lengths and/or *RPLP0* mRNA target sections were compared. No increase in signal intensity was found with two approximately 50 nt long labels when comparing them to a label probe of approximately 30 nt length (Figure S2). To keep a maximum of specificity, the short label probe (Label_33nt) was used in the following experiments. However, identification of a possible trend for well-performing label probes would need the systematic analysis of a larger amount of different labels.

### 3.2. Evaluation of Capture Probe Length

**Figure 2:**
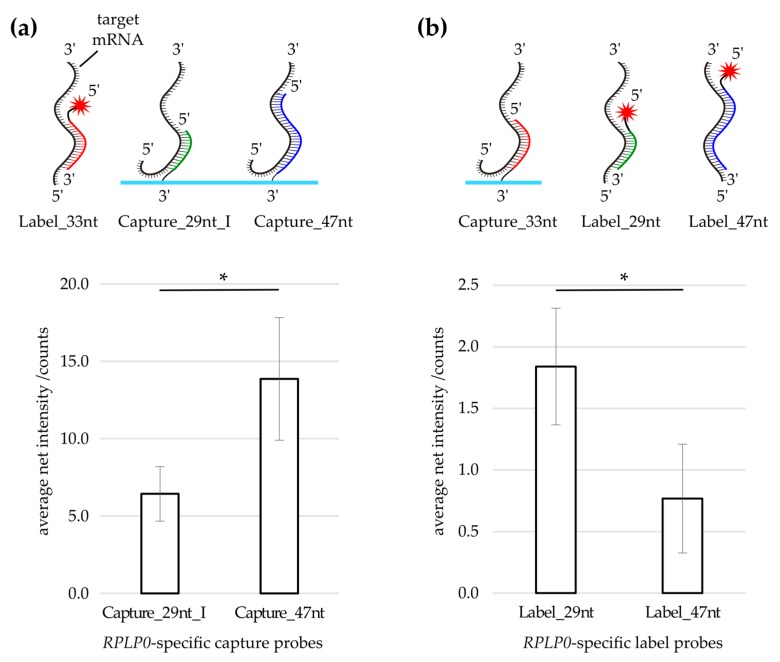
Capture/Label swap: (**a**) Using Label_33nt, the probe Capture_47nt delivered more signal compared to Capture_29nt_I. Bars represent the mean net spot intensities obtained on the *RPLP0*-specific captures depicted on the *x*-axis; (**b**) Upon transforming the former label into a capture (Capture_33nt) and the former captures into labels (Label_29nt, Label_47nt), hybridizations utilizing the elongated Label_47nt did not deliver a higher signal compared to hybridizations performed with Label_29nt. Bars represent the mean net spot intensities obtained on the *RPLP0*-specific capture Capture_33nt; (**a**,**b**) Error bars represent the standard deviation of the mean of three technical replicates. Asterisks indicate the significance level in a one-way ANOVA (* *p* < 0.05).

Next, the effect of specific capture probe elongation was tested by comparing capture probes with different lengths. A comparison of the specific signal obtained on 29 nt (Capture_29nt_I) and 47 nt long capture probes (Capture_47nt) revealed a higher signal on the longer capture (Figure 2a). Similar signal distributions (approximately 70:30 ratio for Capture_47nt) were found with three different labels (Figure S3). To investigate if the higher signal on the 47 nt long capture was due to a better accessibility of the mRNA target section, the modification of the probes used in the initial experiment (Figure 2a) was reversed, *i.e.*, initially 3′-amino-modified probes (captures) were 5′‑Cy5‑modified (labels) and vice versa (Figure 2b). In detail, the label used in the initial experimental setup (Label_33nt) was transformed into a capture (Capture_33nt) and spotted onto a microarray. Furthermore, the former captures (Capture_29nt_I and Capture_47nt) were transformed into label probes (Label_29nt and Label_47nt). As can be seen in Figure 2b, the longer label (Label_47nt) did not deliver a higher signal compared to the shorter label (Label_29nt), which indicates different optimal lengths of label and capture probes, respectively.

### 3.3. Longer Specific Capture Probes Increase Specific Signal

For a further comparison of different capture probe lengths, the 47 nt long capture probe (Capture_47nt) was truncated by 18 nt at its 3′-end to generate the capture probe Capture_29nt. Moreover, Capture_47nt was elongated by 18 nt at its 3′-end to generate Capture_65nt, which was in turn elongated by 28 nt to generate Capture_93nt (Table 2 and S1). As shown in Table 2, all specific elongation steps increased the specific signal. The most prominent signal increase was obtained for the elongation step of the specific capture sequence from 29 nt to 47 nt (7.0 ± 2.6-fold change of the net signal). When comparing fully complementary captures to equally long captures with an unspecific 18 nt or 28 nt long sequence at the 3′ end, the fully complementary captures always delivered a higher signal (Table 2). It was therefore concluded that captures with a longer specific sequence increase the specific signal. Importantly, no trend was found for the unspecific signal on captures with different lengths (Figure S4).

### 3.4. Effect of Capture Probe Spacers

**Figure 3:**
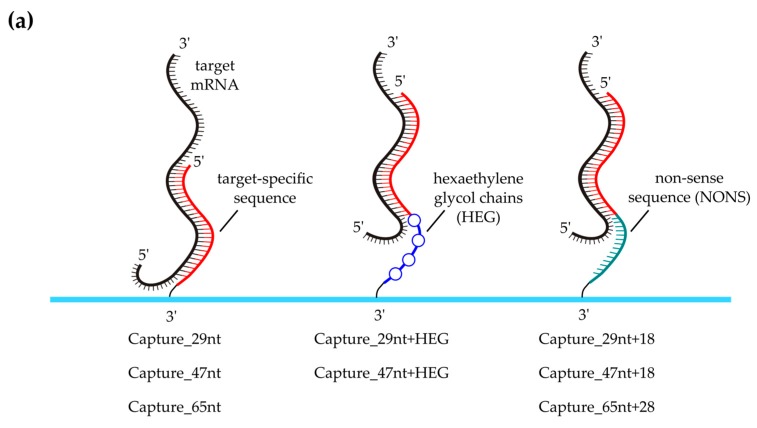

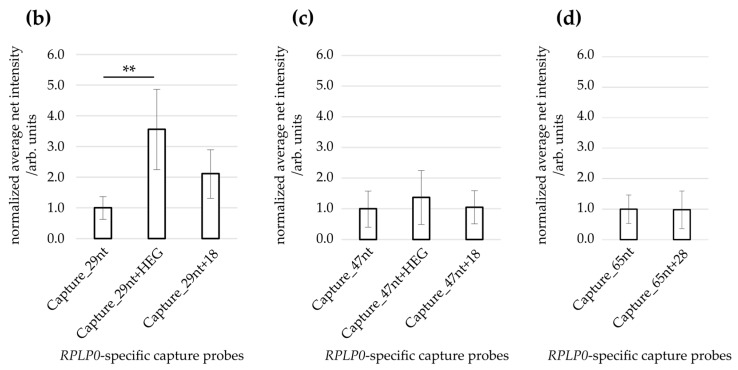
Comparison of capture probes with and without unspecific spacers. (**a**) *RPLP0*‑specific capture probes of different lengths were compared with probes of equal specific length with an additional unspecific 3′ spacer attached. HEG (hexaethylene glycol)-modified capture probes comprised four hexaethylene glycol chains separated by two nucleotides each at their 3′ ends, NONS (non-specific) referred to additional 18–28 non-complementary nucleotides at the 3′ end of the respective capture probe; (**b**) An additional HEG spacer significantly increased the signal on Capture_29nt; (**c**,**d**) The signal on captures with 47 nt or 65 nt specific length could not be increased significantly with additional spacers; (**b**–**d**) Bars represent the mean net spot intensities normalized to the respective *RPLP0*-specific capture without additional spacer, *i.e.*, to Capture_29nt (**b**); Capture_47nt (**c**); or Capture_65nt (**d**). Error bars depict the standard deviation of the mean of five (**b**,**c**) or four (**d**) technical replicates. Asterisks indicate the significance level in a one-way ANOVA (** *p* < 0.01).

It was investigated whether additional spacers between the gene-specific part of the spotted capture probe and the substrate attachment site could further enhance the hybridization efficiency. Therefore, capture probes were tagged at their 3′-ends with either hexaethylene glycol spacers (HEG) or elongated by adding 18 or 28 non-specific nucleotides (NONS), respectively (Figure 3a, Table S1). As shown in Figure 3b–d, the positive effect of spacers decreased with increasing length of the gene-specific capture sequence. Thus, spacers were especially beneficial for the shortest investigated capture probe.

## 4. Discussion

This report shows for the first time the enzyme-free and gene-specific detection of cellular mRNA of a housekeeping gene on a microarray. This was realized by optimizing an innovative double‑hybridization approach previously characterized for detection of synthetic DNA and RNA [22]. *RPLP0* mRNA molecules were labeled by hybridization to complementary DNA label probes with a conjugated 5′-Cy5 fluorophore. Target mRNA was furthermore immobilized on a microarray by hybridization to spotted complementary DNA capture probes. Scanning with an ultra-sensitive fluorescence microscope enabled the highly sensitive detection of mRNA hybridization [22,25,28]. Similar to smFISH techniques, the absence of reverse transcription and amplification steps enabled direct counting of mRNA molecules without introducing enzymatic bias [4,13].

In this publication, we present the design optimization of capture probes used in the direct double‑hybridization analysis of endogenous *RPLP0* mRNA. Tests with *RPLP0*-specific capture probes of different lengths revealed that a target-specific elongation could increase the obtained net signal. Moreover, we found that unspecific spacers located between the gene-specific part of a capture and the substrate attachment site were especially beneficial for the shortest investigated capture probe. This is most likely due to surface effects including electrostatic and steric hindrance, which can reduce the hybridization rate [29,30].

In future experiments, the capture probe optimization presented in this report may also be useful for direct microarray analysis of RNA specimens labeled with alternative methods.

## Figures and Tables

**Table 1:** *RPLP0*-specific label probes used in double-hybridization experiments.

Experiment	*RPLP0* ^a^-Specific Label Probe ^b^				
	Label_29nt	Label_33nt	Label_47nt	Label_51nt	Label_52nt
Capture/Label swap (Figure 2)	10 nM	10 nM	10 nM	–	–
Effect of capture length and spacers (Table 2)	–	10 nM	–	–	–
Spacer effects (Figure 3)	–	10 nM	–	–	–
Label dilution series (Figure S1)	–	100 pM, 1 nM, 10 nM, 100 nM	–	–	–
Label comparison (Figure S2)	–	10 nM	–	10 nM	10 nM
Capture length comparison (Figure S3)	–	10 nM	–	10 nM	10 nM
Unspecific signal with increasing capture length (Figure S4)	–	10 nM	–	–	–

^a^ Large ribosomal protein P0 (*RPLP0*); ^b^ Hybridization mixes comprised additionally one of the listed *RPLP0*-specific label probes in the concentration indicated in the table.

**Table 2:** Pair-wise fold-change on *RPLP0*-specific capture probes.

Compared Capture Probes			Fold-Change ± SD ^a^	*n* ^b^
Original Probe	→	Modified Probe		
Capture_29nt 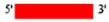	→	Capture_47nt 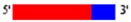	7.0 ± 2.6	5
Capture_47nt 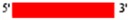	→	Capture_65nt 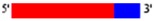	1.9 ± 0.8	9
Capture_65nt 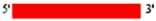	→	Capture_93nt 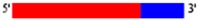	2.0 ± 0.3	4
Capture_29nt+18 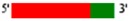	→	Capture_47nt 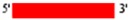	3.2 ± 1.9	5
Capture_47nt+18 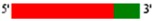	→	Capture_65nt 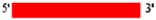	2.3 ± 1.4	5
Capture_65nt+28 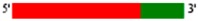	→	Capture_93nt 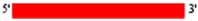	2.0 ± 0.8	4

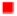
 target-specific capture sequence; 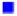 target-specific 3′-elongation; 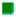 non-specific (NONS) 3′‑elongation; → indicates compared capture probe-pairs; ^a^ For all capture probe-pairs, the fold-change was calculated by dividing the modified probe value by the original probe value; The mean fold-change was obtained by averaging the fold-change values across the technical replicates; SD = Standard deviation of the mean fold-change; ^b^ *n* = Number of technical replicates.

## Supplementary Materials

The following are available online at www.mdpi.com/2076-3905/5/1/5/s1.
