# Supplementary material for: A Double-Hybridization Approach for the Transcription- and Amplification-Free Detection of Specific mRNA on a Microarray
Source: Microarrays (Basel). 2016 Feb 23;5(1):5. doi: 10.3390/microarrays5010005 (PMC5003450; doi:10.3390/microarrays5010005)
Supplement: Supplementary file 1 [file microarrays-05-00005-s001.pdf]

A Double-Hybridization Approach for the Transcription- and Amplification-Free Detection of Specific mRNA on a Microarray

Michaela Haider, Thomas Haselgrübler, Alois Sonnleitner, Fritz Aberger and Jan Hesse

1. Oligonucleotides

The physical properties of the oligonucleotide probes were calculated with OligoAnalyzer 3.1 (IDT DNA, http://eu.idtdna.com). For DNA oligonucleotides >60 nt length, the melting temperature (Tm) was calculated according to the formula:

$$Tm = 67 + 16.6log\frac{[Na^+]}{1+0.7[Na^+]} + 0.8\%GC - \frac{500}{length} - \%Mismatches \quad [31]$$

where by % Mismatches comprised the percentage of nucleotides non-complementary to target mRNA. The influence of formamide on Tm was corrected by subtracting 0.5 °C per percent of formamide from the calculated melting temperature [32]. Capture probes with special modifications were marked by an additional suffix in their name: HEG-modified capture probes comprised four hexaethylene glycol chains separated by two nucleotides each at their 3′ ends. NONS refers to additional 18–28 nucleotides at the 3′-end of a capture probe with no relevant complementarity to the human transcriptome (Table S1).

Table S1. Oligonucleotide sequences.

Name	Sequence
Capture_29nt	5′TCTCCAGTCTTGATCAGCTGCACATCACT–$(CH_2)_7$-amine 3′
Capture_29nt_I	5′GCACATCACTCAGGATTTCAATGGTGCCC–$(CH_2)_7$-amine 3′
Capture_29nt+HEG	5′TCTCCAGTCTTGATCAGCTGCACATCACT[(HEG)AC(HEG)TC(HEG)GT(HEG)GA]–$(CH_2)_7$-amine 3′
Capture_29nt+18	5′TCTCCAGTCTTGATCAGCTGCACATCACT[GACCTAACATGACGGGAC]–$(CH_2)_7$-amine 3′
Capture_33nt	5′TGCAGACAGACACTGGCAACATTGCGGACACCC–$(CH_2)_7$-amine3′
Capture_47nt	5′TCTCCAGTCTTGATCAGCTGCACATCACTAGGATTTCAATGGTGCC–$(CH_2)_7$-amine 3′
Capture_47nt+HEG	5′TCTCCAGTCTTGATCAGCTGCACATCACTCAGGATTTCAATGGTGCC[(HEG)AC(HEG)TC(HEG)GT(HEG)GA]–$(CH_2)_7$-amine 3′
Capture_47nt+18	5′TCTCCAGTCTTGATCAGCTGCACATCACTCAGGATTTCAATGGTGCC[GACCTAACATGACGGGAC]–$(CH_2)_7$-amine 3′
Capture_65nt	5′TCTCCAGTCTTGATCAGCTGCACATCACTCAGGATTTCAATGGTGCCCCTGGAGATTTTAGTGGT–$(CH_2)_7$-amine 3′
Capture_65nt+28[a]	5′AGTCTTGATCAGCTGCACATCACTCAGGATTTCAATGGTGCCCCTGGAGATTTTAGTGGTGATAC[GACCTAACATGACGGGACGACCTAACAT]–$(CH_2)_7$-amine 3′
Capture_93nt[a]	5′AGTCTTGATCAGCTGCACATCACTCAGGATTTCAATGGTGCCCCTGGAGATTTTAGTGGTGATACCTAAAGCCTGGAAAAAGGAGGTCTTCTC–$(CH_2)_7$-amine 3′
Control(+) capture	5′CTGCAGGTCTGGCCTTTGAGTGGTGAAATC–$(CH_2)_7$-amine 3′
Control(+) label	5′ Cy5-$(CH_2)_6$-GCTTCATCCTGTGTCTCATAATCTGGGCGT 3′
Control(+) RNA	5′GAUUUCACCACUCAAAGGCCAGACCUGCAGACGCCCAGAUUAUGAGACACAGGAUGAAGC3′
Control1(-)	5′GTCTCCATTTCACCTGTGCATATGAGCTG–$(CH_2)_7$-amine 3′
Control2(-)	5′TTGAACCAGTTGTTAAATTCTTTGCCATTTG–$(CH_2)_7$-amine 3′
Label_29nt	5′ Cy5-$(CH_2)_6$-GCACATCACTCAGGATTTCAATGGTGCCC3′
Label_47nt	5′ Cy5-$(CH_2)_6$-TCTCCAGTCTTGATCAGCTGCACATCACTCAGGATTTCAATGGTGCC3′
Label_51nt	5′ Cy5-$(CH_2)_6$-GTTGGGTAGCCAATCTGCAGACAGACACTGGCAACATTGCGGACACCCTCC 3′
Label_52nt	5′ Cy5-$(CH_2)_6$-AGCACTTCAGGGTTGTAGATGCTGCCATTGTCGAACACCTGCTGGATGACCA 3′

Label_47nt	5′ Cy5-$(CH_2)_6$-TCTCCAGTCTTGATCAGCTGCACATCACTCAGGATTTCAATGGTGCC3′

[a] Note that the targeted mRNA section of Capture_65nt+28 and Capture_93nt was shifted by five nucleotides compared to Capture_29nt(+HEG,+18), Capture_47nt(+HEG,+18) and Capture_65nt to avoid unfavorable secondary structures of the capture molecule.

2. *RPLP0* Label Dilution Series

The double-hybridization assay was based on a recently published report dealing with the detection of short DNA and RNA oligonucleotides on a microarray with single-molecule sensitivity [22]. In the presented work, the assay conditions were modified to enable the detection of endogenous mRNA molecules. Therefore, 50% (*v*/*v*) deionized formamide was added to the hybridization mix to decrease the melting temperature of mRNA secondary structures. As the number of *RPLP0* mRNA molecules in the applied 100 ng/µL isolated total RNA was unknown, a suitable label concentration had to be determined first. Therefore, a *RPLP0* label dilution series was conducted with 100 pM–100 nM Label_33nt probe concentration while keeping the mRNA concentration constant. As shown in Figure S1, the specific *RPLP0* signal increased with increasing label concentration from 100 pM to 10 nM. A further increase to 100 nM label concentration could not enhance the signal any more. Moreover, the signal on the non-complementary capture probes Control1(-) and Control2(-) remained low and stable across the whole label concentration range. For the following probe characterization, a label concentration of 10 nM was used in all experiments comprising 100 ng/µL isolated total Panc-1 RNA.

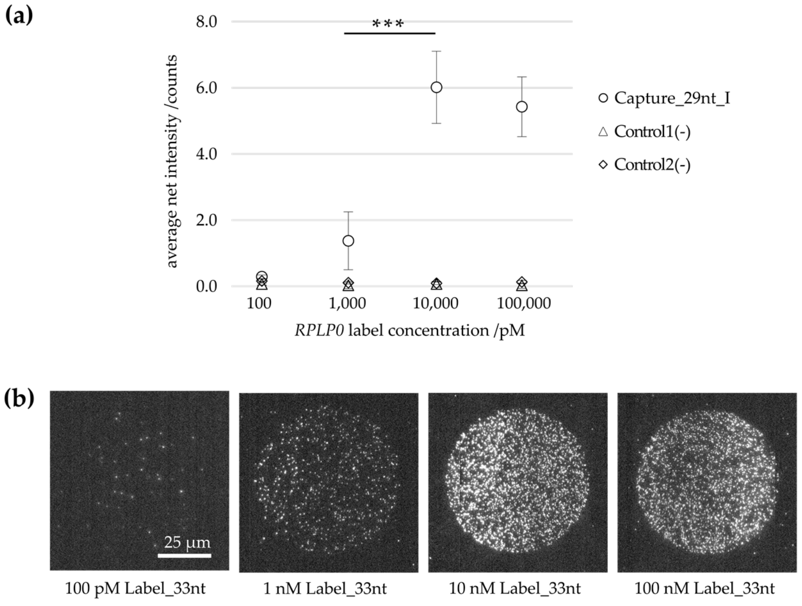

Figure S1. *RPLP0* label probe dilution series. (**a**) To determine a suitable label concentration for sensitive *RPLP0* detection, a label dilution series was conducted. The concentration of Label_33nt was therefore increased from 100 pM to 100 nM while keeping the target concentration constant. Open circles represent the mean net spot intensities obtained on the *RPLP0*-specific capture Capture_29nt_I, open triangles represent the mean net signals obtained on the non-specific capture Control1(-), and open diamonds represent the mean net signals on the non-specific capture Control2(-). Error bars depict the standard deviation of the mean of 4–5 technical replicates. Asterisks indicate the significance level in a one-way ANOVA (*** $p < 0.001$); (**b**) Fluorescence images obtained on microarray spots hybridized with constant target concentration and increasing label concentration. The signal increased with increasing label concentration up to 10 nM. A further increase to 100 nM did not increase the specific signal any more.

3. Assessment of Different *RPLP0* Label Probes

As shown in Figure S2a, the probe Label_33nt was elongated at both ends with *RPLP0*-complementary nucleotides to create Label_51nt. The third probe, termed Label_52nt, targeted a different section of the *RPLP0* mRNA. No increase in signal intensity was found with longer labels when compared to the *RPLP0* signal obtained with Label_33nt (Figure S2b). Thus, the initially used label probe, Label_33nt, was retained for further experiments. However, to identify a possible trend for preferential lengths of label probes, the systematic analysis of multiple different label probes would be required.

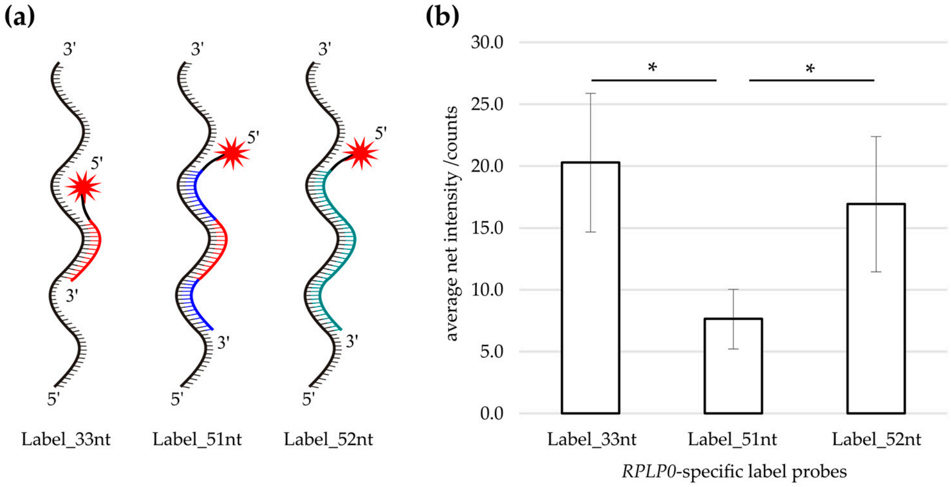

Figure S2. Signal strength with different *RPLP0* label probes. (**a**) Three different *RPLP0*-specific label probes were compared. The shortest probe, Label_33nt (red), was elongated at both ends with *RPLP0*-complementary nucleotides (blue) to generate Label_51nt. Label_52nt (green) was of similar length as Label_51nt but targeted another section of the *RPLP0* mRNA; (**b**) No increase in specific signal intensity was found with longer label probes. Bars represent the summarized net signals obtained on the *RPLP0*-specific captures Capture_29nt_I and Capture_47nt. Error bars depict the standard deviation of the mean of 3–4 technical replicates. Asterisks indicate the significance level in a one-way ANOVA (* $p < 0.05$).

4. *RPLP0* Capture Length Comparison with Different Label Probes

RPLP0-specific capture probes with different lengths (Capture_29nt_I and Capture_47nt) were compared using three different *RPLP0*-specific label probes (Label_33nt, Label_51nt, and Label_52nt, Figure S2a). With all labels, the longer capture (Capture_47nt) delivered approximately 70% of the total *RPLP0* signal per microarray (that is, the sum of specific signal obtained on Capture_29nt_I and Capture_47nt) (Figure S3).

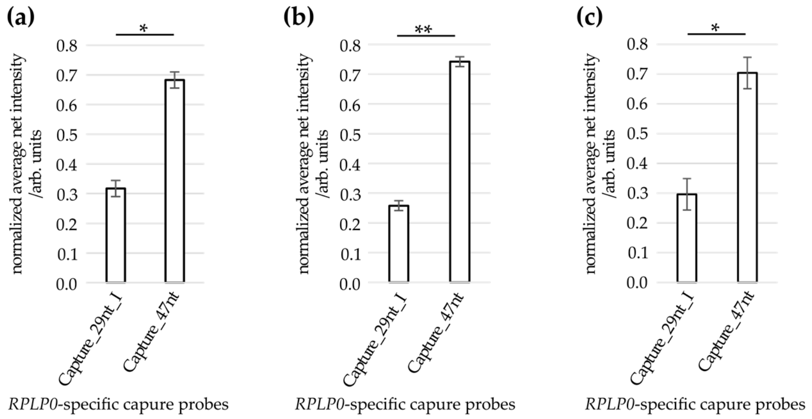

Figure S3. Signal strength on different *RPLP0* capture probes. The signal distribution on two *RPLP0*-specific captures with different lengths was investigated using three different label probes:

Label_33nt. (**a**); Label_51nt (**b**); and Label_52nt (**c**). All labels delivered a similar signal distribution of approximately 70:30 in favor of the longer capture. Bars represent the mean net spot intensities normalized to the total *RPLP0*-specific net signal per microarray. Error bars represent the standard deviation of the mean of 3–4 technical replicates. Asterisks indicate the significance level in a one-way ANOVA (* $p < 0.05$, ** $p < 0.01$).

5. Unspecific Signal on Different Capture Probes

The unspecific signal consisting of spot autofluorescence and direct binding of Label_33nt to *RPLP0*-specific and control capture probes was compared. As can be seen in Figure S4, the unspecific signal did not increase with increasing capture length. Thus, the specificity was maintained for the investigated long (*i.e.*, >50 nt length) capture probes.

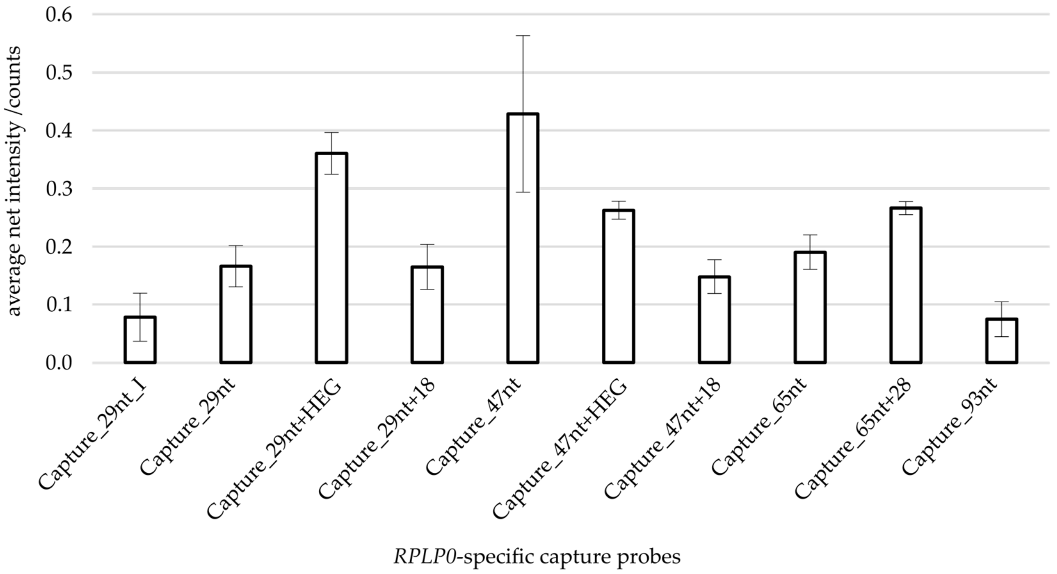

Figure S4. Unspecific signal on different *RPLP0*-specific capture probes. The unspecific signal on different capture probes was compared. No trend was found when comparing the unspecific signal and the capture length. Bars represent the mean net spot intensities obtained with the *RPLP0*-specific captures indicated on the *x*-axis. Error bars represent the standard error of the mean of 4–7 technical replicates.

References

31. Wetmur, J.G. DNA probes: Applications of the Principles of Nucleic acid Hybridization. *Crit. Rev. Biochem. Mol. Biol.* **1991**, *26*, 227–259.
32. Baumgart, E.; Schad, A.; Grabenbauer, M. *In situ* hybridization: General Principles and Application of Digoxigenin-Labeled cRNA for the Detection of mRNAs. In *Immunocytochemistry and In Situ Hybridization in the Biomedical Sciences*; Birkhäuser: Boston, MA, USA, 2001; pp. 108–137.
